# Caucasian Infants Scan Own- and Other-Race Faces Differently

**DOI:** 10.1371/journal.pone.0018621

**Published:** 2011-04-13

**Authors:** Andrea Wheeler, Gizelle Anzures, Paul C. Quinn, Olivier Pascalis, Danielle S. Omrin, Kang Lee

**Affiliations:** 1 Dr. Eric Jackman Institute of Child Study, University of Toronto, Toronto, Canada; 2 Department of Psychology, University of Delaware, Newark, Delaware, United States of America; 3 Laboratoire de Psychologie et NeuroCognition, Université Pierre Mendès, Grenoble, France; 4 Department of Psychology and Center for Human Development, University of California San Diego, La Jolla, California, United States of America; Kyushu University, Japan

## Abstract

Young infants are known to prefer own-race faces to other race faces and recognize own-race faces better than other-race faces. However, it is entirely unclear as to whether infants also attend to different parts of own- and other-race faces differently, which may provide an important clue as to how and why the own-race face recognition advantage emerges so early. The present study used eye tracking methodology to investigate whether 6- to 10-month-old Caucasian infants (N = 37) have differential scanning patterns for dynamically displayed own- and other-race faces. We found that even though infants spent a similar amount of time looking at own- and other-race faces, with increased age, infants increasingly looked longer at the eyes of own-race faces and less at the mouths of own-race faces. These findings suggest experience-based tuning of the infant's face processing system to optimally process own-race faces that are different in physiognomy from other-race faces. In addition, the present results, taken together with recent own- and other-race eye tracking findings with infants and adults, provide strong support for an enculturation hypothesis that East Asians and Westerners may be socialized to scan faces differently due to each culture's conventions regarding mutual gaze during interpersonal communication.

## Introduction

In recent years, one of the most heavily investigated topics within face processing research has been the differential processing of own- and other-race faces. The popularity of this topic can be attributed to multiple factors including the fact that the topic contributes to our understanding of the relationship between experience and visual information processing [1]. Research in this area also has the potential to contribute to a more complete understanding of highly controversial and complex phenomena such as the origin and development of racial prejudices and stereotypes [2], [3].

The differential processing of own- and other-race faces has been termed the other-race effect and has been found to exist in adults [4], [5], children [1], [6]–[12], and infants [1], [13]–[17]. Within the adult literature, the other-race effect is most often described in terms of the ability to recognize own-race faces more quickly and easily than other-race faces. Several hypotheses have been proposed to account for the recognition effect, among which the contact hypothesis has received the most attention [5], [18]. This hypothesis suggests that extensive experience with own-race faces and a relative lack of experience with other-race faces leads to better processing for own-race faces than other-race faces.

Prior to 2000, only a handful of developmental studies had examined differences in own- and other-race face processing in children and infants [6], [7]. In the last five to six years, much has been learned about the early emergence of race-dependent face processing differences. Sangrigoli and de Schonen [11] used the size of the inversion effect produced by own- and other-race faces to index the other-race effect in 4- and 5-year-olds, with the rationale that a greater inversion effect for own-race than other-race faces would suggest a greater expertise in processing own-race faces. They reported that children demonstrated a larger inversion effect when processing own-race faces as opposed to other-race faces. That is, 4- to 5-year-olds were better able to recognize upright own-race faces as compared to inverted own-race faces, but showed no difference in their processing of upright and inverted other-race faces. Such findings provide indirect evidence for the existence of an other-race effect during childhood. Additionally, an own-race recognition advantage has been directly observed with kindergarten-aged children [10] and children between 8 and 16 years of age [9], [12].

Complementing the above research with children, recent findings have also been accumulating on the emergence of the other-race effect in infancy. Kelly et al. [19], [20] and Bar-Haim, Ziv, Lamy, and Hodes [21] reported that infants as young as 3 months of age demonstrate a preference to attend to own-race faces over other-race faces, but newborns did not show such a preference [19]. Other research with infants suggests that with increased age, when infants from various racial backgrounds view faces from their respective racial/ethnic groups, they become better able to discriminate or recognize own-race faces [13], [15]–[17]. Also, a perceptual narrowing phenomenon has been observed: Whereas 3-month-olds are able to recognize own-race faces and faces from various other races, with increased age, they become less capable of recognizing other-race faces. By 9 months of age, they can only recognize own-race faces. Furthermore, Anzures, Quinn, Pascalis, Slater, and Lee [22] found that other-race effects extend beyond face preference and recognition. They reported that 9-month-olds can form a category of other-race faces within which faces are not differentiated at the individual level, reflecting a form of categorical perception. In contrast, for own-race faces, 9-month-olds' categorization is further differentiated to the individual level, reflecting a genuine form of categorization.

Thus, experience with own- versus other-race faces plays an important role in infants' preference, discrimination, and categorization for faces as early as the first few months of life. This body of literature tells us *what* infants and children do when processing own- and other-race faces and provides evidence as to *when* this phenomenon begins to emerge. However, what remains unclear is *how* and *why* the other-race effect develops. Most of the studies examining the other-race effect in infancy have relied on paired preference paradigms and measures of overall looking time. The designs typically rely on off-line coding of infants' eye movements to determine which one of two paired stimuli infants prefer to examine. Such measures are naturally coarse: they can only provide a global level of analysis of infants' visual attention. They cannot determine whether the specific aspects of own- and other-race faces are attended to by infants differently, which would provide a more fine-grained understanding of the emergence of the other-race effect in infancy.

To the best of our knowledge, only one eye-tracking study has been conducted with infants to date, which examined similarities and differences in visual scanning of own- and other-race faces. Liu et al. [23] presented 4- to 9-month old Chinese infants living in China with dynamic videos of both Caucasian and Chinese face stimuli while eye-tracking data were collected. The authors reported that with increased age, the Chinese infants fixated significantly less on the noses of the Caucasian faces: Their fixation on the noses of the Chinese faces did not change with age. There were no significant age-related changes with regard to fixations on the eyes or mouth for either the own- or the other-race faces.

These findings by Liu et al. [23] are intriguing in light of the recent findings by Blais, Jack, Scheepers, Fiset, and Caldara [24]. They found that East-Asian adults tended to fixate on the nose region of faces when scanning own-race faces, differing from the fixation patterns produced by Caucasian adults that focus strongly on the eyes. In addition, they found that Asians and Caucasians would generalize their own-race scanning patterns to other-race faces but the patterns were not as robust as for own-race faces. Blais et al. [24] hypothesized that their findings suggested that face scanning patterns are not universally pre-determined but rather are shaped by the observer's culture. Liu et al. [23] also speculated that in early infancy such culture-specific scanning patterns, if any exist, may be engendered initially by the specific facial physiognomy of the infants' own race, with which they become increasingly familiarized. Indeed, anthropometric studies of facial morphology between different racial groups have revealed marked differences [25]–[27]. For example, noses of Asian faces tend to be wider but shorter than noses of Caucasian faces. Thus, Liu et al. [23] speculated that the observer race should interact with the target face race in influencing infants' scanning patterns of own- and other-race faces. Because they only tested Chinese children with Chinese and Caucasian faces, this hypothesis needs to be tested with infants from racial backgrounds other than Chinese, as well as with target faces from other races.

To address this issue directly, the present study collected eye-tracking data from Caucasian infants while viewing own- and other-race faces. We aimed to examine whether infants would show different eye tracking patterns when they viewed own- and other-race faces. As recent research has shown that Caucasian infants aged 6 months do not display a strong other-race effect when tested on Chinese and Caucasian faces [15], a somewhat more distinct other-race face group was selected. African-American/Black faces have been shown to illicit strong other-race effects from early on in development, in that both Chinese and Caucasian infants are unable to discriminate them past 3 months of age [15], [16]. Also, Hajnis, Farkas, Ngim, Lee, and Venkatadri [26] showed significant facial morphological differences among Caucasian and Black adult faces. For example, Black adult faces have significantly wider noses and mouths than Caucasian adult faces; however, both have similar eye regions. Infants in the present study were therefore shown dynamic videos of Caucasian and Black adult female faces while eye-tracking data were collected. We specifically focused on infants' fixations of the internal facial features, namely, eyes, nose, and mouth, which are perceptually the high-contrast regions of faces that are important for recognition [28], and which are also thought to convey substantial social information for interpersonal communication [29]. Additionally, the analyses of fixation data on these features ensured direct comparisons with the recent eye-tracking studies involving infants [23] and adults [24].

## Methods

### Participants

In total, 56 infants were recruited. Infants were of Caucasian descent and were recruited through mailers sent to parents in the community. All parents indicated that the infants had no regular exposure to Black faces. Among the infants, 19 infants were excluded due to failure to complete the calibration procedure (n = 5), incomplete data capture (n = 5), or because parents were non-Caucasian or mixed race (n = 9). The final sample consisted of 37 infants (*Mean Age* = 236.85 days, *Standard Deviation* = 35.69 days, age range: 184 days—300 days, 25 males [68%]).

The present study was conducted in accordance to the Tri-Council Research Ethics Guidelines. The Office of Research Ethics at the University of Toronto approved the experimental procedure and the informed consent protocol. Written informed consents were obtained from the infants' parents prior to their participation in the study.

### Materials

Stimuli were comprised of six videos of adult females (three Caucasian and three Black) looking directly into a camera with a neutral expression and counting upwards for 30 seconds. All video recordings were made in front of a uniform light-colored background and were presented without sound. Adult females were the mothers of infants who had participated in a previous study. Female, rather than male adult faces, were chosen because existing studies have shown that female adult faces are seen far more frequently than male adult faces and children tend to be more receptive to female adult faces than to male ones [1].

### Procedure

Mothers were informed of the purpose of the study and gave written consent for their child to participate. Infants were secured in a car seat and placed in a semi-reclined position (approximately 45 degrees) beneath a 21-inch Tobii 2150 eye tracker with a sampling rate of 50 Hz and a screen resolution of 800×600. The eye tracking screen was positioned at an angle parallel to the incline of the infant, about 60 cm from the infant's eyes. An experimenter sat directly behind the infants to adjust the car seat as required during the calibration procedure and to reorient the infants' gaze if the infants were inattentive for more than 3 seconds. Infants were first shown an attention grabbing video of a toy with accompanying audio in order to orient their attention towards the display. Infants then completed a calibration procedure in which two alternating toys appeared at five locations across the screen: the four corners, and the centre (calibrations courtesy of Scott Johnson). If insufficient data were collected to complete the calibration task, it was repeated up to three times for a total of four attempted calibrations.

Infants were then presented with two 30-second video clips on the eye-tracking screen while fixation data were captured. The stimulus faces were 21.0×14.1 degrees of visual angle on average. Each infant saw one own-race face and one other-race face. The particular female exemplar from each race was chosen randomly, and the order of the two videos was counterbalanced across infants.

### Data Analysis

Data were mainly analyzed for the total duration of fixations in milliseconds within an area of interest relative to the total on-face looking time for each condition (see below). Fixations were defined as having a minimum radius of 30 pixels and a minimum duration of 100 ms.

**Figure 1:**
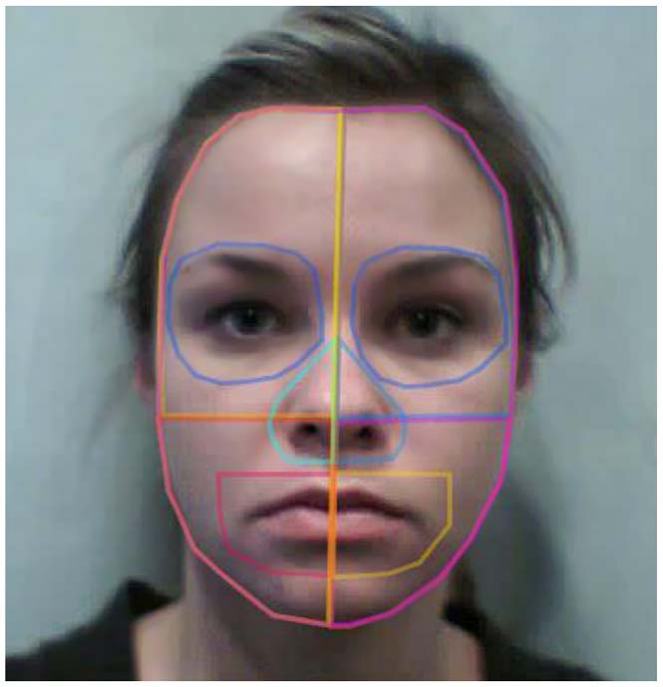
Example Areas of Interest (AOI) plots (Consent and permission from the model have been obtained).

Because the purpose of the present study was to examine whether infants fixate on different parts of the own- and other-race faces differently or similarly, we first created three Areas of Interest (AOIs) for the eyes, nose, and mouth (see Figure 1 for an example) by outlining them with a small buffer area to allow for feature and head movements during the recording. The buffer zone for the nose and eyes was approximately 0.5 cm (18.9 pixels), while the buffer zone for the mouth was extended to approximately 1.0 cm (37.8 pixels) to allow for some slight movements when the female models talked. Further, we created two additional AOIs by dividing the face into an upper part and a lower part using a line across the center of the nose and the lower edges of the ears (see Figure 1). Adding the fixation time for the upper and lower AOIs would yield the total on-face looking time.

## Results

Preliminary analyses of variances on the factors of infant gender and stimulus order failed to show significant effects of the order of conditions and infants' genders. The data for these two factors were thus combined for the subsequent analyses.

### Overall Duration of Entire Stimulus and Face Fixation

Table 1 shows the means and standard deviations of the total on-stimulus fixation time and total on-face looking time. The difference between the on-stimulus and on-face fixations was that the on-face fixations only included fixations on the face itself as defined by combining the fixation times on the upper and lower face AOIs. In contrast, the on-stimulus fixations included both the on-face fixations as well as the fixations outside the face AOIs that were still within the computer monitor screen. In other words, the on-stimulus fixations included possibly the fixations on the target's hair and neck, the background, and even the fixations within the face contour. As can be seen in Table 1, the mean total on-stimulus time and on-face time were virtually identical, indicating that during the experiments, infants mainly focused on the face, not other parts of the screen. However, because the total video length was 30 seconds, it can be deduced that about half of the time, the infants either looked away or looked at the screen, or their fixation length did not reach the 100 ms threshold to be counted as a fixation.

**Table 1:** Means and standard deviations of total on-stimulus and on-face time (seconds) in the own-race and other-race conditions.

	Total on-stimulus fixation time		Total on-face fixation time	
	Other-race faces	Own-race faces	Other-race faces	Own-race faces
**Mean**	14.78	15.69	14.21	15.12
**Std. Deviation**	6.99	8.13	6.89	7.74

Two analyses of variance were first conducted to determine if there was an overall preference to attend to one face type over the other. A repeated measures 2 (stimulus race: own vs. other)×1 (age in days: continuous) ANOVA was performed with stimulus race (own or other) as a within-subjects variable, participant age as a continuous variable, and total on-stimulus or on-face fixation duration in seconds as the dependent variable. The reason to use age as a continuous variable rather than dividing the participants into age groups was not only because the former has greater statistical power than the latter, but also because using age as a continuous variable allows for capturing age-related changes in eye tracking behaviors at a finer time scale (in days). Dividing participants into age groups would unnecessarily lose such additional fine-grained age differences that the precision of eye tracking data affords.

For the on-stimulus fixation time, only the main effect of age was significant, *F*(1,35) = 17.27, *p*<.0001, η*_ṗ_^2^ = *.33. To determine this significant effect, Pearson correlations were calculated between the infants' ages in days and the total on-stimulus fixation time for the other- and own-race conditions, *r(37)* = −.44, *p* = .007, and *r(37)* = −.59, *p*<.0001, respectively. With increased age, the total on-stimulus fixation time decreased significantly. Similar to the on-stimulus fixation time, for the on-face fixation time, only the age main effect was significant, *F*(1,35) = 15.34, *p*<.0001, η*_p_^2^ = *.31. To determine this significant effect, Pearson correlations were calculated between the infants' ages in days and the total on-face fixation time for the other- and own-race conditions, *r(37)* = −.40, *p* = .014, and *r(37)* = −.57, p<.0001, respectively. With increased age, the total on-face fixation time also decreased significantly.

### Proportional Fixation of Individual AOIs

Next, the fixation data were converted into proportional fixation times within each AOI relative to the total on-face fixation times for each condition. The resulting data set thus included proportional fixation duration scores for each of the AOIs. The reason to convert the AOI fixation time data into proportions was that we were mainly interested in whether infants had different patterns of fixations on different face areas and major face features of the own- and other-race faces. Because the participants at different ages were already different in their total on-face fixation times, their fixation times on each AOI would naturally be different, which in turn would make it difficult to determine whether infants at various ages also had the differential fixation patterns on different parts of the face. Thus, to address our research question adequately, we needed to adjust participants' fixation times on each face feature by their total fixation time on the whole face. Table 2 shows the means and standard deviations of the proportions of fixation time on each of the major AOIs of the own- and other-race faces.

**Table 2:** Means (SD) of proportion of fixation time on each AOI for own- and other-race faces.

AOI	Other-race face	Own-race face
Upper	.60 (.26)	.62 (.29)
Lower	.40 (.26)	.38 (29)
Eyes	.29 (.21)	.34 (.23)
Nose	.13 (.11)	.20 (.22)
Mouth	.23 (20)	.20 (.18)
Upper (minus mouth/nose)	.25 (.20)	.18 (.19)
Lower (minus nose/mouth)	.10 (.15)	.08 (.19)

#### Upper and lower AOIs

To explore whether infants attended to the upper parts of the own- and other-race faces differentially, we conducted a 2 (race: own versus other)×1 (age in days: continuous variable) repeated measures ANOVA on the proportions of fixation time on the upper AOIs of the own- and other-race faces. The main effects of age and race were significant, *F*(1,35) = 4.21, *p* = .048, η*_ṗ_^2^ = *.11, and *F*(1,35) = 9.33, *p* = .004, η*_ṗ_^2^ = *.21, respectively, which was modified by a significant two-way interaction, *F*(1,35) = 9.88, *p* = .003, η*_ṗ_^2^ = *.22. To explore this significant interaction, we calculated Pearson correlations between age in days and infants' proportions of fixation time on the upper parts of the own- and other-race faces. The age in days was only significant with the fixation time on the upper part of the own-race faces, *r(37)* = .50, *p* = .002, but not with that for the upper part of the other-race faces, *r(37)* = .05, *p* = .750. Thus, as infants became older, their fixation time on the upper part of the own-race faces increased, whereas their fixation time on the upper part of the other-race faces did not change with age.

We also performed a similar analysis on the lower AOIs and the results were mirror images of the above analysis, which was expected because the proportions for the upper and lower AOIs should add up to 100%. Thus, with increased age, infants' fixation times on the lower part of the own-race faces became smaller, whereas their fixation times on the lower part of the other-race faces did change.

#### Eyes, nose, and mouth AOIs

We examined infants' visual attention to the three major face features, specifically the eyes, nose, and mouth, because they are high-contrast regions of faces that carry important information for face identity [28] as well as for interpersonal communication [29]. A 2 (race: own versus other)×3 (feature: eyes, nose, mouth)×1 (age in days: continuous variable) repeated measures ANOVA was performed using the proportion of fixation time on eyes, nose, and mouth as the dependent variable and age in days as a continuous variable. Only the main effect of age was significant, *F*(1,35) = 5.59, *p* = .024, η*_ṗ_^2^ = *.14. The feature X race and age X feature effects were significant or marginally significant, respectively, *F*(1,70) = 3.57, *p* = .033, η*_ṗ_^2^ = *.09, and *F*(2,70) = 3.12, *p* = .050, η*_ṗ_^2^ = *.08. These significant two-way interactions were modified by a significant three-way interaction between race, feature, and age in days, *F*(2,70) = 4.29, *p* = .018, η*_ṗ_^2^ = *.11. No other effects were significant.

**Figure 2:**
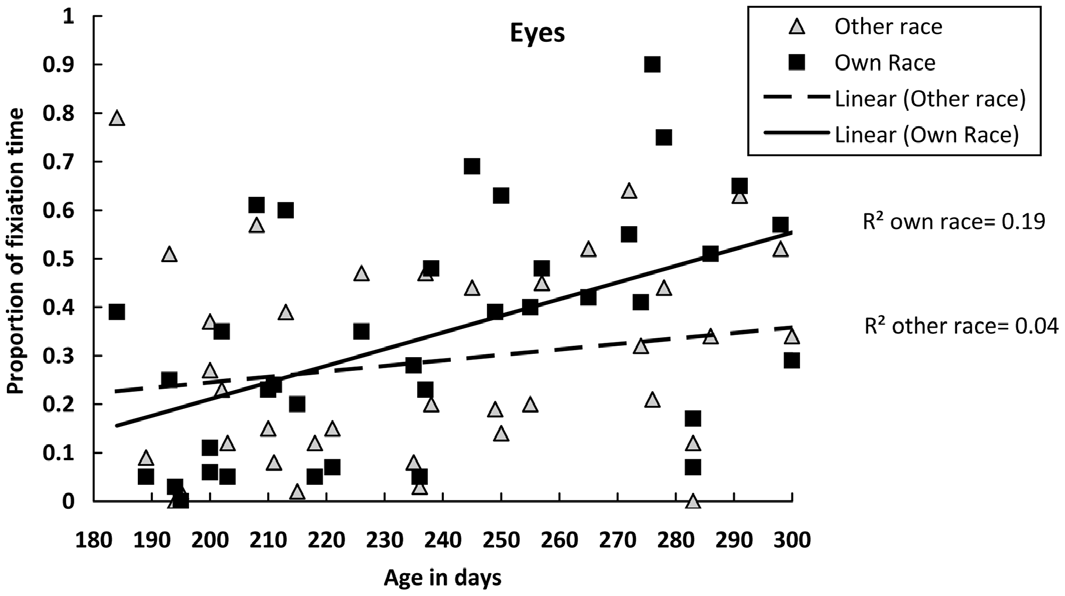
Proportion of time spent fixating the eyes of the own- and other-race faces as a function of age in days.

To further explore this significant three-way interaction, we calculated Pearson correlations between the infants' ages in days and individual proportion scores of fixation time on either the other- or own-race eyes. The age in days was significantly and positively correlated with the proportion of fixation time on the own-race eyes (*r(37)* = .51, *p* = .001), but not with other-race eyes (*r(37)* = .19, *p* = .259). Thus, with increased age, infants spent an increasingly greater amount of the time fixating on the eyes of own-race faces, whereas the proportion of fixation time on the eyes of other-race faces remained largely unchanged with age (Figure 2).

**Figure 3:**
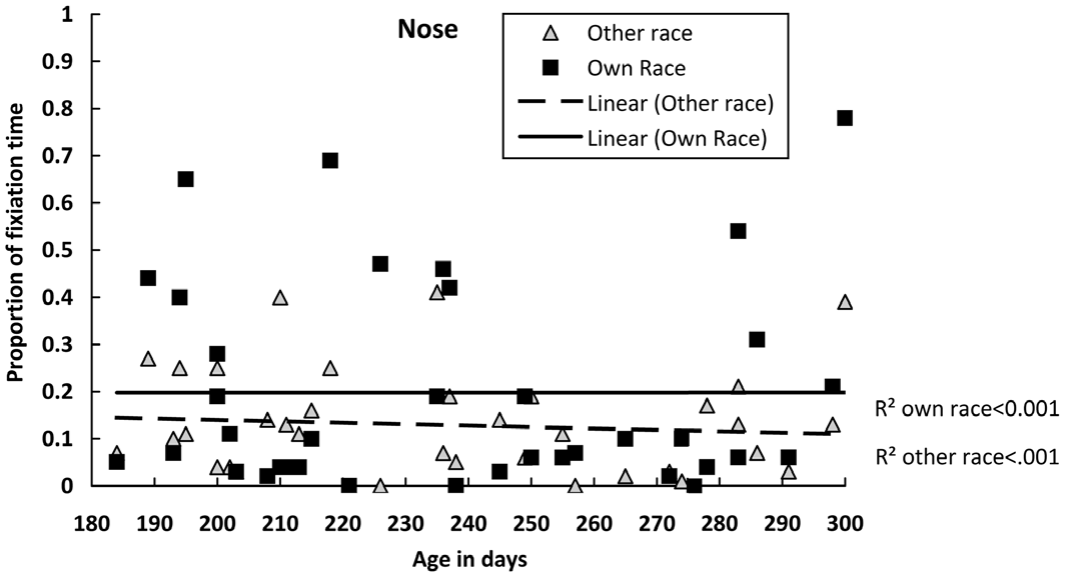
Proportion of time spent fixating the nose of the own- and other-race faces as a function of age in days.

For the proportion of fixation time on the nose, we calculated Pearson correlations between the infants' ages in days and the proportion of fixation time on either the other- or own-race nose, respectively. The age in days was not significantly correlated with the proportion of fixation time on the nose of either the other- or own-race faces, *r(37)* = −.10, *p* = .573, and *r(37)* = .00, *p* = .999. Thus, infants' fixation times on the nose of both races remained unchanged with age (Figure 3).

**Figure 4:**
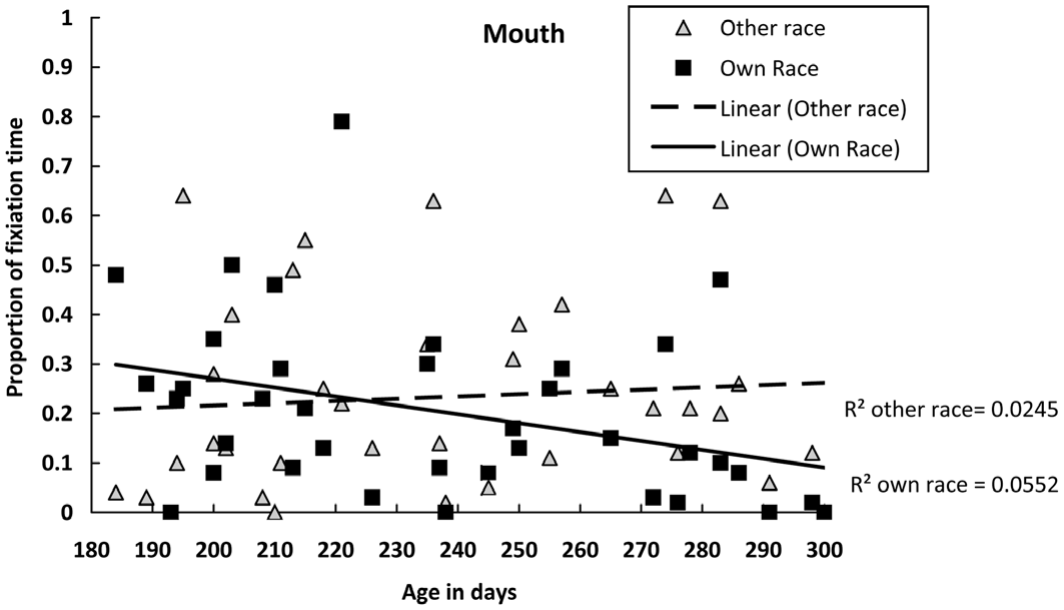
Proportion of time spent fixating the mouth of the own- and other-race faces as a function of age in days.

For the proportion of fixation time on the mouth, we calculated Pearson correlations between the infants' ages in days and the proportion of fixation time on either the other- or own-race mouth, respectively. The age in days was significantly and negatively correlated with the proportion of fixation time on the mouth of the own-race faces, *r(37)* = −.34, *p* = .029, but not with the fixation time on the mouth of the other-race faces, *r(37)* = .08, *p* = .623. Thus, with increased age, infants became less inclined to look at the own-race mouth, whereas their fixation time on the other-race mouth remained unchanged (Figure 4).

We also analyzed the proportions of on-face fixations that did not fall within the three major face feature AOIs (Table 2). No significant cross-race effects and their related effects were found.

## Discussion

The present study investigated the visual attention of infants to faces belonging to their own race and faces of another race with the use of the eye tracking methodology. We specifically aimed to examine whether infants, with increased age, would show differential visual attention to different parts of own- and other-race faces.

Consistent with the Chinese infants in Liu et al. [23], Caucasian infants overall spent a similar amount of time fixating on the own-race faces as compared to the other-race faces. With increased age, infants' total fixation times on both own- and other-race faces decreased significantly, likely due to the fact that the face stimuli, though dynamic, were silent and not interactive [30]. With increased age, infants might become easily habituated to, and therefore look away from the stimulus, resulting in a reduction in total on-stimulus and on-face fixation times.

However, when we considered the proportions of fixation time on the faces specifically, other-race effects in terms of visual fixation patterns emerged. Further, the other-race effects also increased with age. As age increased, infants became more inclined to fixate on the upper portion of the own-race faces, whereas their fixations on the same part of the other-race faces remained unchanged. This age-related other-race effect was attributable to infants' differential fixations on the eyes of the own- and other-race faces. We found that as age increased, infants' fixations were more or less the same for the eyes of the other-race faces, but they became more inclined to attend to the eyes of the own-race faces. In contrast, their looking times at the nose was not affected by either age or face race.

Mirroring the other-race effect regarding the fixation time on the upper parts of the own- and other-race faces, we found that infants became less inclined to attend to the lower parts of the own-race faces with increased age. On the other hand, their visual attention to the lower parts of the other-race faces remained similar. This other-race effect in the lower portion of the face was clearly due to the fact that infants became less inclined to attend to the mouth of the own-race faces with increased age, whereas their visual attention to the mouth of other-race faces did not change with increased age.

The above other-race effects in visual scanning patterns among Caucasian infants should be compared with those of Liu et al. [23] who tested Chinese infants between 4 and 9 months of age and found seemingly different results. In Liu et al. [23], when the full age range was considered, Chinese infants' visual attention to the nose of the other-race Caucasian faces decreased significantly with age, whereas their visual attention to the nose of the own-race Chinese faces did not change with age. However, intriguingly, when we reanalyzed their data by only including infants who were older than 6 months of age so as to make it comparable to the participant age range of the present study, with increased age, Chinese infants significantly increased their visual attention to the nose of the own-race Chinese faces, whereas their visual attention to the nose of the other-race Caucasian faces decreased significantly. These fixation patterns on the noses of the own- and other race faces differed from the results of the present study. Also different from our findings was that Chinese infants maintained the same level of visual attention to the eyes and mouth of the own-race (Chinese) and other-race (Caucasian) faces.

One could attribute the differences in outcomes of the two studies to the nature of the own- and other-race faces used. The infants in Liu et al. [23] viewed other-race Caucasian faces and own-race Chinese faces. Alternatively, in the present study, the other-race faces were Black and the own-race faces were Caucasian. Certain race-specific facial features inherent in Chinese, Black, and Caucasian faces might have driven infants, regardless of their own race, to attend to the face features differently. This suggestion is supported by evidence from anthropometric studies of facial morphology between Asian, Black, and Caucasian adults [25]–[27] who reported major cross-race differences in craniofacial characteristics. When compared with Caucasian faces, Black faces have significantly wider noses and mouths, but both races have similar eye regions. In contrast, relative to Caucasian faces, Chinese faces have a wider distance between the inner corners of the eyes but a smaller eye width, a wider nose, and a smaller mouth width. These unique facial morphological features might have led infants in Liu et al. [23] and those in the present study to scan the faces of different races differently.

However, this possibility alone cannot explain why infants in Liu et al. [23] and those infants in our study scanned the Caucasian faces differently. If the unique facial morphology of the Caucasian faces alone drives infants' visual attention, Chinese and Caucasian infants in both studies should have attended to Caucasian eyes with increased age, but in fact only Caucasian infants did so. Clearly, experience with a certain face race may also play an important role in infants' scanning patterns. As of now, it remains to be determined exactly how experience shapes an individual's visual attention patterns towards a class of faces. Additional empirical work is needed to address this question involving infants, children, and adults. In infancy, further studies should compare Chinese infants' processing of Chinese and Black faces to Caucasian infants' processing of Caucasian and Black faces. The commonalities and differences in processing faces of different races from such data should elucidate the specific roles of face experience and facial cranial characteristics of a particular face race in the development of the other-race effects seen in the present study and in Liu et al. [23].

It should be noted that although the findings from both the present study and Liu et al. [23] differed from one another, when taken together, they are actually highly consistent with the enculturation hypothesis proposed by Caldara and his colleagues [24], [31], [32]. This hypothesis suggests that through cultural learning, one develops a culture-specific manner of face scanning whereby Asians focus on the nose of the target face regardless of its race and Caucasians focus on the eyes of the target face, also regardless of the face race. This hypothesis, as mentioned above, was supported by the findings of Blais et al. [24]. They found that East-Asian adults tended to fixate on the nose region of faces when scanning own-race faces, whereas Caucasian adults tended to focus strongly on the eyes. Also, Asians and Caucasians generalized their own-race scanning patterns to other-race faces although the patterns were not as robust as for own-race faces. Kelly, Miellet, and Caldara [32] replicated the central findings of Blais et al. [24], but also found that the culture-specific scanning pattern extends beyond human faces to monkey faces and to non-face yet nevertheless face-like Greebles. Further, Caldara, Zhou, and Miellet [31] suggested that Asian adult observers' nose-centric scanning patterns may be part of their strategy to rely on peripheral vision to code facial information. They found that Asian participants' scanning patterns would show similarities to Caucasian scanning patterns when they were prevented from using their peripheral vision to extract face identity information. Nevertheless, when their field of vision was not restricted, they readily reverted to the typical nose-centric scanning pattern. Kelly et al. [32] speculated that the culture-specific scanning patterns may stem from the social norms concerning gaze avoidance and engagement when interacting with others. Indeed, the existing studies about gaze and mutual gaze in Asia and the West have not only found differences in mutual gaze behaviors but also revealed Asians' proclivity to avoid attending to the eyes of another person to show politeness or respect [33]. This cross-cultural difference in mutual gaze has been found to emerge even in early infancy [34].

Although the enculturation hypothesis was proposed to account for the robust cross-race differences in visual scanning patterns in Caucasian and Asian adult observers, it may be applicable to Chinese and Caucasian infants and children. Both our study and that by Liu et al. [23] suggest that Caucasian infants indeed appear to develop towards a visual attention pattern that focuses on the eyes, whereas Chinese infants appear to develop towards a visual attention pattern that focuses on the nose. One inconsistency between the enculturation hypothesis and the existing infant data is that the apparent culture-specific visual attention patterns of Chinese and Caucasian infants appear to be specific only to the faces of their own race. Moreover, the hypothesis predicts observer culture-specific scanning patterns that are independent of face race. One possibility is that the culture-specific pattern of visual scanning is developed initially to achieve an optimal level of processing of the own-race faces that one encounters most frequently. Once this scanning pattern is well established, it may become automatically deployed to process faces in general, including other-race faces and even non-human faces or face-like visual objects [32].

This idea would be consistent with the suggestion by Liu et al. [23], such that distinct features may be differentially useful in recognizing faces of different races. Such a possibility would be consistent with Valentine's [35], [36] account of how the other-race effect comes about in face space (i.e., the tuning of the face processing system to features that maximize discrimination of same-race faces but not necessarily other-race faces). It would also be consistent with the findings of cross-race differences in cranial face morphology [27]. These observations suggest that a full account of other-race face-processing may require an understanding of both stimulus and observer contributions operating during the short and long terms of development. The short-term factors include morphology differences in faces from different races that could be detected by infants during the first year of life, as well as the infants' experiences with faces from different races. Thus, it is necessary to carry out additional studies that for instance replicate the present study with Caucasian infants. Other-race Asian faces could be used as stimuli, and African American infant participants can be recruited to view own-race faces, and Caucasian and Chinese other-race faces. Moreover, the long-term factors include the facial morphologies diagnostic for expert-level face processing that optimize the differentiations among own-race faces [37], and children's culture-specific conventions regarding interpersonal gaze interactions. Further, we need to examine how adults in different cultures interact with infants and whether the differential face-to-face interaction behaviors indeed lead infants to scan different parts of own-race faces in a systematic manner.

Lastly, given that the current research revealed significant differences in the visual attention of infants to own- and other-race faces, future research should aim to combine eye-tracking data collection with tests of discrimination, recognition, and categorization of faces of different races in both infants and children. Such research would contribute to a more complete understanding of the effect of differential scanning patterns for own- and other-race faces, and eventually provide an account of how and why other-race effects in face processing emerge, develop, and reach an adult level.
